# SNH-119014, a novel pyruvate kinase activator, enhances ATP production and reduces oxidative stress in erythroid cells from patients with β-thalassemia major

**DOI:** 10.3389/fphar.2026.1719328

**Published:** 2026-04-23

**Authors:** Qiulin Huang, Yumei Huang, Lingyuan Pan, Rongrong Liu, Yongrong Lai

**Affiliations:** 1 Department of Hematology, The First Affiliated Hospital of Guangxi Medical University, Nanning, Guangxi, China; 2 NHC Key Laboratory of Thalassemia Medicine, Nanning, China; 3 Guangxi Key Laboratory of Thalassemia Research, Nanning, China; 4 Key Laboratory of Hematology, Education Department of Guangxi Zhuang Autonomous Region, Guangxi Medical University, Nanning, Guangxi, China

**Keywords:** adenosine triphosphate, anemia, glycolysis, metabonomics, pyruvate kinase, thalassemia

## Abstract

**Background:**

Thalassemia is a common disease worldwide. Oxidative stress contributes to ineffective erythropoiesis and hemolysis in β-thalassemia major (β-TM). Increasing cellular adenosine triphosphate (ATP) production by activating the activity of the pyruvate kinase (PK) may counteract oxidative stress. SNH-119014 is a novel allosteric PK activator targeting the same protein as mitapivat (AG-348), a first-in-class agent known to ameliorate anemia in thalassemia. This study aim to evluate the effects of SNH-119014 in erythroid cells from β-TM patients.

**Methods:**

PK activity was measured in recombinant human PKLR isoforms incubated with a range of concentrations of SNH-119014. Peripheral blood samples were obtained from 30 β-TM patients and 30 healthy controls (HCs), and the isolated red blood cells (RBCs) were assessed for ATP content and analyzed by metabolomics. Bone marrow samples were collected from 14 β-TM patients and four healthy volunteers, and CD34^+^ hematopoietic stem and progenitor cells (HSPCs) were isolated. HSPCs were differentiated into erythroid precursors. Erythroid precursors were treated with 5 μM SNH-119014, 5 µM AG-348, or vehicle, followed by assessment of ATP content, reactive oxygen species (ROS) levels, the reduced/oxidized glutathione ratio, and metabolomic profiling.

**Results:**

SNH-119014 activated PK with an AC_50_ of 28.90 nM and was characterized by a higher maximal efficacy (Emax = 949.9%) compared to AG-348 (AC_50_ = 13.71 nM; Emax = 749.4%). Consistently, in RBCs from β-TM patients, SNH-119014 increased ATP by a mean of 129% compared to 131% for AG-348 (p = 0.0928). Based on metabolomics, KEGG pathway analysis revealed that glycolysis/gluconeogenesis and the pentose phosphate pathway were among the top ten most significantly enriched pathways for both SNH-119014 and AG-348. Considering ineffective erythropoiesis is a important factor contributor to thalassemia pathology, we further investigated the effects of SNH-119014 in erythroid precursors. SNH-119014 increased ATP levels, reduced ROS, and improved the GSH/GSSG ratio in erythroid precursors.

**Conclusion:**

In conclusion, SNH-119014 activates PK, thereby increasing ATP production and alleviating oxidative stress in erythroid cells from β-TM patients. **S**NH-119014 exhibits a promising pharmacological profile, supporting its further development as a potential therapy for thalassemia.

## Introduction

Maintaining erythropoiesis and red blood cells (RBCs) function requires substantial energy (Park et al., 2010). Lacking mitochondria, RBCs depend on glycolysis to generate adenosine triphosphate (ATP), which is essential for sustaining cellular metabolism, membrane integrity, and defense against oxidative stress (McMahon et al., 2021; van Dijk et al., 2023). Thalassemia is prevalent inherited disorder worldwide (Rachmilewitz and Giardina, 2011). The pathogenesis of β-thalassemia is caused by reduced ynthesis of β-globin and a consequent excess of α-globin chains. These free α-globin induces oxidative stress, leading to apoptosis of erythropoiesis and hemolytic anemia (Ginzb et al., 2011; Voskou et al., 2015; Longo et al., 2021). Moreover, clearing excess α-globin imposes a significant additional demand on ATP in erythroid cells. Therefore, efficient ATP generation is crucial for preserving erythroid cell function and membrane integrity.

Mitapivat (AG-348) is a first-in-class allosteric activator of pyruvate kinase (PK) (Alayash, 2021). Preclinical studies have demonstrated that AG-348 increases PK activity and elevates cellular ATP content in erythroid cells (Kung et al., 2017). Studies in thalassemia mouse models further showed that AG-348 ameliorated anemia by reducing hemolysis and ineffective hematopoiesis (Mattè et al., 2023; Matte et al., 2021). Building on these preclinical benefits, AG-348 has advanced to clinical trials, with ongoing phase 2 (NCT03692052) (van Dijk et al., 2022) and phase 3 studies (ENERGIZE for non-transfusion-dependent thalassemia, NCT04770753; ENERGIZE-T for transfusion-dependent α or β-thalassemia, NCT04770779) for thalassemia (Cappellini et al., 2024; Kattamis, 2022; Musallam et al., 2022; Al-Samkari and van Beers, 2021).

The investigational compound SNH-119014 (C_20_H_19_F_2_N_3_O_4_S) is a bis(azanylylidene) sulfonyl derivative, with the systematic name (S)-2-(2,4-difluorophenyl)-3-hydroxy-1-(1'-(pyridin-2-ylsulfonyl)-1′,4′-dihydro-2H,2′H-[3,3′-biazetylidene]-1(4H)-yl)propan-1-one, which acts as an allosteric activator of PK. This study began with an evaluation of SNH-119014s activation of human PK using molecular docking and *in vitro* enzymology assays. Subsequently, the cellular ATP content was tesed and a metabolomics analysis of RBCs from healthy volunteers and β-TM patients was applied. Subsequently, the influence of SNH-119014 on glycolytic flux and other metabolic pathways was assessed from the metabolomic data. Finally, the effect of SNH-119014 in erythroid precursor cells from β-TM patients was investigated.

## Methods

### Molecular docking

**FIGURE 1:**
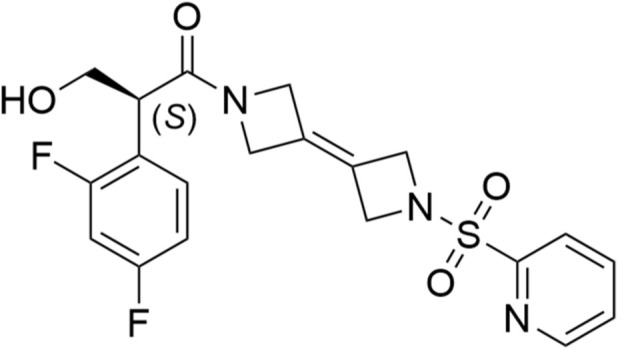
Structure of SNH-119014.

The binding of SNH-119014 and AG-348 (Kung et al., 2017) to PKLR and PKM2 was assessed by molecular docking. The crystal structures of PKLR (PDB: 8XFD Chain A, B), and PKM2 (PDB: 8G2E) were obtained from the RCSB Protein Data Bank. Proteins were prepared using the Protein Preparation Wizard module in Schrödinger Maestro. The chemical structure of SNH-119014 is shown in Figure 1. Molecular docking was performed using the Glide XP mode to dock each ligand into the active site of the respective protein. Subsequently, the binding free energy was estimated by molecular mechanics generalized born surface area (MM-GBSA) calculations.

### Measurement of pyruvate kinase activity

Pyruvate kinase (PK) activity was measured using a commercial kit (Pyruvate Kinase Assay Kit, Abcam). Recombinant PKLR isoforms (R&D Systems) were incubated with a range of concentrations of SNH-119014 or AG-348, and activity was quantified following the manufacturer’s protocol (Zhao et al., 2023).

### *Ex vivo* treatment of RBC

Peripheral blood samples were obtained from healthy controls (HCs) and patients with β-thalassemia major (β-TM) under a protocol approved by the Ethics Committee of The First Affiliated Hospital of Guangxi Medical University (Approval No.: 2023-E322-01). All patients were on regular transfusion and iron chelation therapy (e.g., deferasirox), and blood was drawn at least 3 weeks post-transfusion. All samples were processed within 2 hours of collection.

Leukocyte-depleted RBCs were isolated by percoll density gradient centrifugation and resuspended in phosphate-buffered saline supplemented with 1% glucose, 170 mg/L adenine, and 5.25 g/L mannitol (AGAM solution) (Schroeder et al., 2022; Liu et al., 2023; Rab et al., 2019). Based on preliminary dose-and time-response experiments (Supplementary Tables S1–S4), the optimal incubation conditions for RBCs were determined to be 10 μM of compounds for 4 h, as ATP levels reached a plateau at concentrations ≥10 μM and incubation times ≥4 h. Following incubation, intracellular ATP levels were measured using the CellCounting-Lite 2.0 Luminescent Cell Viability Assay (Vazyme Biotech). For metabolomic analysis, a total of 120 samples were prepated, comprising four groups: untreated β-TM RBCs, β-TM RBCs incubated with SNH-119014, β-TM RBCs incubated with AG-348, and untreated HCs RBCs. Metabolite identification was performed using the R package in conjunction with the BiotreeDB (version 3.0) (Zhou et al., 2022). Both ATP and metabolomic profiling were carried out on RBCs derived from independent donations.

### *In vitro* treatment of erythropoiesis

Bone marrow aspirates were obtained from 14 β-TM patients and four healthy volunteers. CD34^+^ hematopoietic stem and progenitor cells (HSPCs) were isolated from bone marrow mononuclear cells, which were first separated by Percoll density gradient centrifugation, using anti-CD34 antibody-conjugated magnetic beads (Miltenyi Biotec).

For expansion, cells were cultured for 6 days in Serum-Free Expansion Media (StemCell Technologies) supplemented with StemSpan™ CC100 (StemCell Technologies), erythropoietin (3 IU/mL MedChemExpress), and 1% penicillin-streptomycin (1%, Gibco) (Hu et al., 2013; Lessard et al., 2015; Sankaran et al., 2008). Subsequently, cells were reseeded and maintained in differentiation media consisting of Serum-Free Expansion Media supplemented with the following cytokines and factors: erythropoietin (3 IU/mL, MedChemExpress), dexamethasone (2 μM, Yuanye Bio-Technology), interleukin-3 (5 ng/mL; Novoprotein), stem cell factor (20 ng/mL; Novoprotein), β-estradiol (1 μM; Yuanye Bio-Technology), and penicillin-streptomycin (1%, Gibco). Cells were treated with 5 μM AG-348, 5 μM SNH-119014, or vehicle control, based on dose-response data (Supplementary Figure S4). Compounds were added to the culture medium on days 7, 9, 11, and 13 of differentiation.

To assess differentiation status, erythroid precursors were analyzed by flow cytometry using fluorochrome-conjugated antibodies from BD Biosciences: PE-anti-CD71, APC-anti-CD235a (glycophorin A), and FITC-anti-CD34. Analysis was performed on a FACSVerse™ flow cytometer (BD Biosciences), and data were processed with FlowJo™ software (v10.8.1; BD Biosciences). Apoptosis, reactive oxygen species (ROS) levels, and the glutathione (GSH/GSSG) ratio were assessed using an Annexin V-PE detection kit (Multi Sciences), the CM-H_2_DCFDA probe (10 μM; MedChemExpress), and a commercial assay kit (Beyotime Biotechnology), respectively, following the manufacturers’ protocols (Giustarini et al., 2013).

### Statistical analysis

The normality of data distribution was assessed using the Shapiro-Wilk test. For normally distributed data, comparisons were made using paired or unpaired Student’s t-tests; otherwise, non-parametric tests were applied. A p-value <0.05 was considered statistically significant.

## Results

### Docking results

**FIGURE 2:**
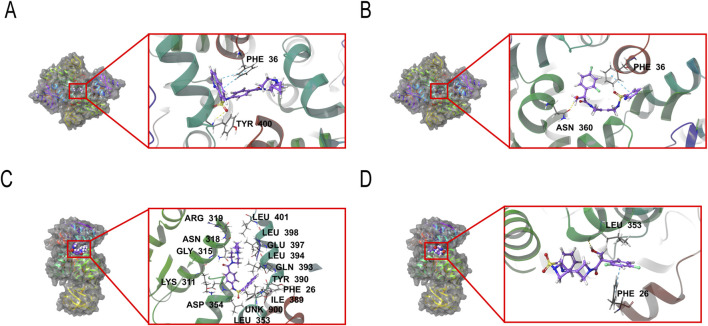
Molecular docking of SNH-119014 and AG-348 to pyruvate kinase isoforms. **(A)** Molecular docking of AG-348 bound to PKLR. **(B)** Molecular docking of SNH-119014 bound to PKLR. **(C)** Molecular docking of AG-348 bound to PKM2. **(D)** Molecular docking of SNH-119014 bound to PKM2.

The binding modes and affinities of SNH-119014 and AG-348 were evaluated by molecular docking and MM-GBSA calculations. Docking poses generated in XP precision mode were primarily assessed using the XP Gscore, where a value lower than −6.0 typically indicates favorable ligand-protein binding stability (Dong and Li, 2024). Complementarily, the MM-GBSA method was employed to estimate the binding free energy (MM-GBSA dG Bind). A dG Bind value more negative than −30 kcal/mol is considered indicative of strong binding and supports the formation of a stable complex (Dong and Li, 2024; Wang et al., 2023; Friesner et al., 2006; Teng et al., 2023). For PKLR, AG-348 showed an XP Gscore of −7.573 and an MM-GBSA dG Bind of −49.94 kcal/mol (Figure 2A). SNH-119014 exhibited an XP Gscore of −5.016 and a dG Bind of −42.61 kcal/mol for the same target, suggesting stable binding (Figure 2B). For PKM2, AG-348 had a docking score of −3.594 with a dG Bind of −40.78 kcal/mol (Figure 2C), whereas SNH-119014 showed a score of −2.756 and a dG Bind of −38.56 kcal/mol (Figure 2D).

### SNH-119014 enhances PKLR activity and increases ATP levels in RBCs

**FIGURE 3:**
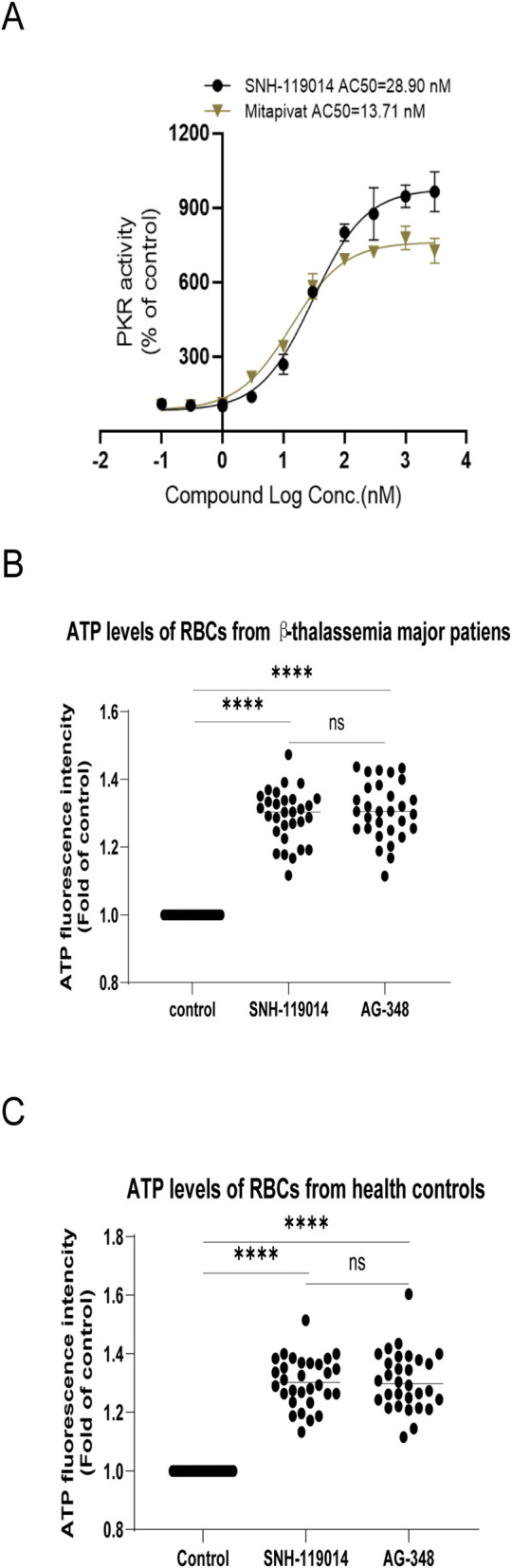
SNH-119014 increases the activity of PKLR and ATP levels in Red Blood Cell (RBC) to an extent comparable to AG-348. **(A)** The activity of the recombinant PKLR enzyme incubated with indicated concentrations of SNH-119014. Experiments were performed in triplicate. **(B)** SNH-119014 increases ATP levels in RBCs of β-thalassemia major to an extent comparable to AG-348. **(C)** SNH-119014 increases ATP levels in RBCs of healtht conrols to an extent comparable to AG-348. Note: Experiments of each sample were performed in triplicate. Data are mean. **** indicates p < 0.0001.

Dose-response assays demonstrated that SNH-119014 and AG-348 concentration-dependently activated recombinant human PKLR. SNH-119014 exhibited a concentration for 50% of maximal activity (AC_50_) of 28.90 nM, a maximal efficacy (Emax) of 949.9%, and a Hill coefficient of 1.243. AG-348 showed a lower AC_50_ (13.71 nM) and E_max_ (749.4%), with a Hill coefficient of 1.200 (Figure 3A).

The glycolysis pathway is subjected to a complex mechanism of inhibiting and stimulating factors (Van Wijk and Van Solinge, 2005). Therefore, experiments were conducted to test whether glycolytic metabolism in RBCs is promoted by SNH-119014. Thirty patients with β-TM and 30 healthy volunteers were enrolled in this study (Table 1). In β-TM RBCs, ATP increased to a comparable level after incubating with SNH-119014 or AG-348 [mean increase: 129% (range: 112%–147%) vs. 131% (range: 119%–143%), p = 0.0928] (Figure 3B). A similar increase was observed in RBCs from healthy volunteers [130% (range: 113%–151%) vs. 131% (range: 112%–160%), p = 0.7553] (Figure 3C).

**TABLE 1:** Characteristics of patients with β-thalassemia major and healthy volunteers.

Variable	Patients with β-thalassemia major (n = 30)	Healthy volunteers (n = 30)
Age, year, Median (interquartile range)	18 (12, 22)	26 (24, 33)
Male, n (%)	22 (73.3%)	14 (46.7%)
Women, n (%)	8 (26.7%)	16 (53.3%)
Hemoglobin (g/L)	84 (81,91)	139 (120,149)
Reticulocyte (×10^9^/L)	81 (36,140)	54 (34,74)
Genotype, n (%)	​	Not applicable
β^41-42^/β^41-42^	5	​
β^41-42^/β^17^	5	​
β^654^/β^17^	5	​
β^41-42^/β^−28^	5	​
β^654^/β^654^	2	​
β^17^/β^17^	2	​
β^654^/β^−28^	1	​
β^−28^/β^17^	1	​
β^71-72^/β^−28^	1	​
β^17^/β^18^	1	​
β^654^/β^17^	1	​
β^654^/β^17^	1	​

### Metabolomic profile of RBCs

**FIGURE 4:**
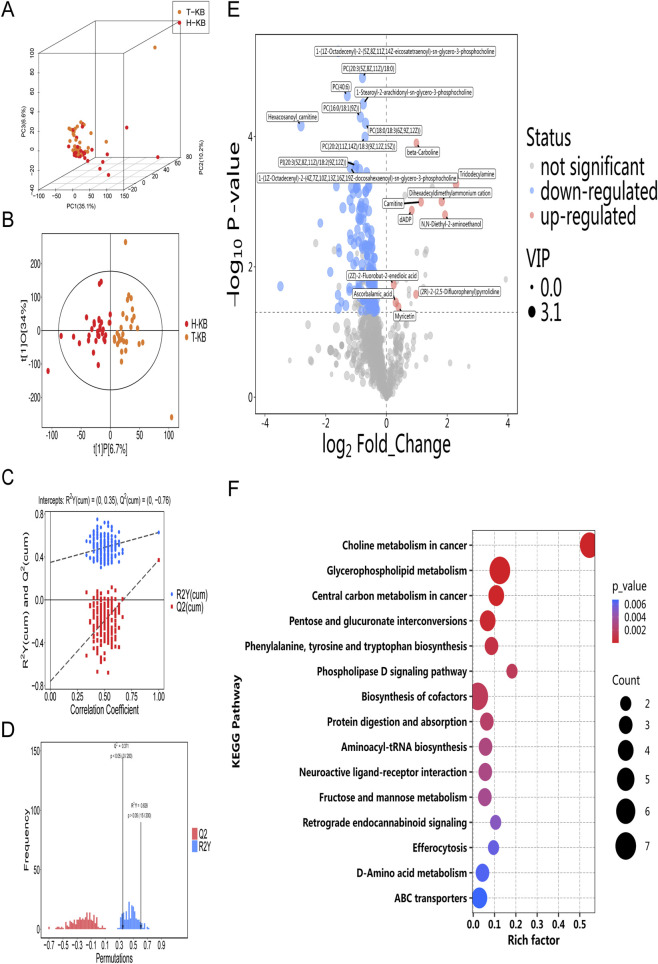
Metabolic profiling of red blood cells (RBCs) from β-thalassemia major (β-TM) patients and healthy controls. **(A)** Principal component analysis (PCA) of RBC between 30 β-TM (T-KB) patients and 30 healthy controls (H-KB). **(B)** Supervised orthogonal projections to latent structures discriminant analysis (OPLS-DA) of RBC between 30 β-TM (T-KB) patients and 30 healthy controls (H-KB). R^2^X = 0.407 (cum), R^2^Y = 0.628 (cum), Q^2^ = 0.371 (cum). **(C)** Permutation test validation of the OPLS-DA model discriminating between 30 β-TM (T-KB) patients and 30 healthy controls (H-KB). **(D)** Histogram of permutation test for the OPLS-DA model comparing between 30 β-TM (T-KB) patients and 30 healthy controls (H-KB). **(E)** Volcano plot of differentially abundant metabolites between 30 β-TM (T-KB) patients and 30 healthy controls (H-KB). To clear visualization, the top 10 significantly upregulated and top 10 downregulated metabolites were labeled. The dot size was proportional to the VIP value, with larger dots representing higher VIP scores. Significantly upregulated, downregulated, and non-significant metabolites are depicted in red, blue, and gray, respectively. **(F)** KEGG enrichment analysis of differentially abundant metabolites between 30 β-TM (T-KB) patients and 30 healthy controls.

To compare the metabolic profiles of RBCs from β-TM patients and healthy volunteers, untargeted metabolomics was performed. Principal component analysis (PCA) revealed limited separation between the metabolic profiles of β-TM patients and healthy volunteers (Figure 4A). In contrast, supervised orthogonal partial least squares-discriminant analysis (OPLS-DA) clearly distinguished the two groups (Figures 4B–D).

We defined differential metabolites as those satisfying both a p-value <0.05 (Student’s t-test) and a VIP score>1 in this study. A total of 181 differential metabolites were identified between red blood cells from β-TM patients and healthy controls (HCs). Among these, glycerophospholipids constituted the most abundant category (n = 86, 47.5%). The volcano plot in Figure 4E displays the top 10 significantly up- and downregulated metabolites. Further enrichment of differential metabolites is presented in Figure 4F.

### SNH-119014 reprograms the metabolism of RBCs

**FIGURE 5:**
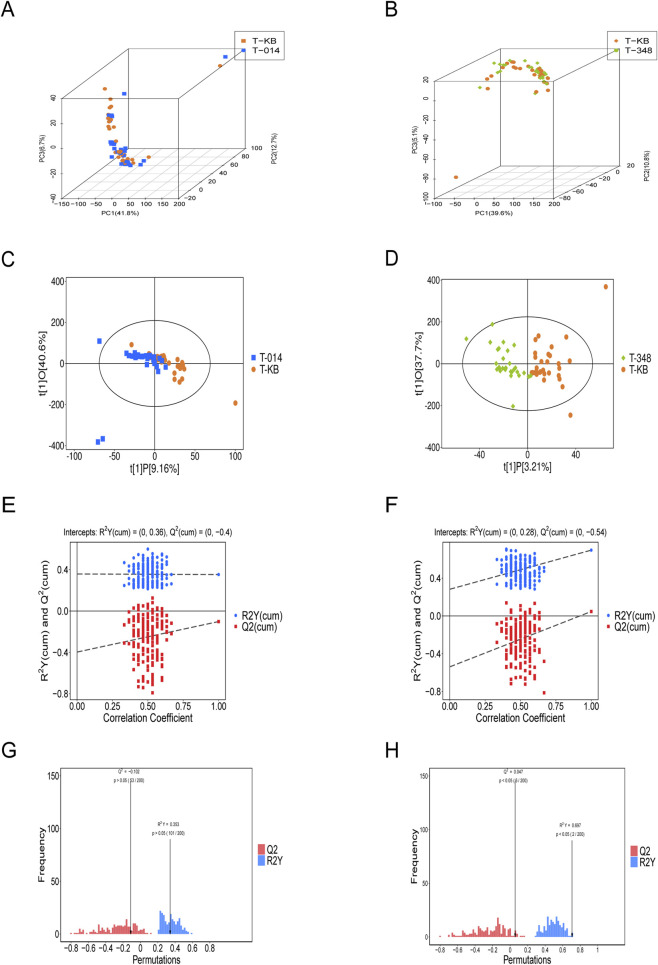
Metabolic reprogramming of β-thalassemia major (β-TM) red blood cells (RBCs) by SNH-119014 and AG-348. **(A,B)** Principal component analysis (PCA) score plots of RBCs from β-TM patients at baseline (T-KB) versus after incubation with SNH-119014 (T-014) or AG-348 (T-348), respectively. **(C,D)** Supervised orthogonal projections to latent structures discriminate analysis (OPLS-DA) of RBCs between 30 β-TM (T-KB) patients and incubated with SNH-119014 (T-014) or AG-348 (T-348). T-KB vs. T-014: R^2^X = 0.497 (cum), R^2^Y = 0.353 (cum), Q^2^ = −0.102 (cum). T-KB vs. T-348: R^2^X = 0.409 (cum), R^2^Y = 0.697 (cum), Q^2^ = 0.0473 (cum). **(E,F)** Permutation tests (200 iterations) validating the OPLS-DA models shown in **(C,D)**, respectively. **(G,H)** Histograms of permutation test results for the models in **(C,D)**, respectively.

**FIGURE 6:**
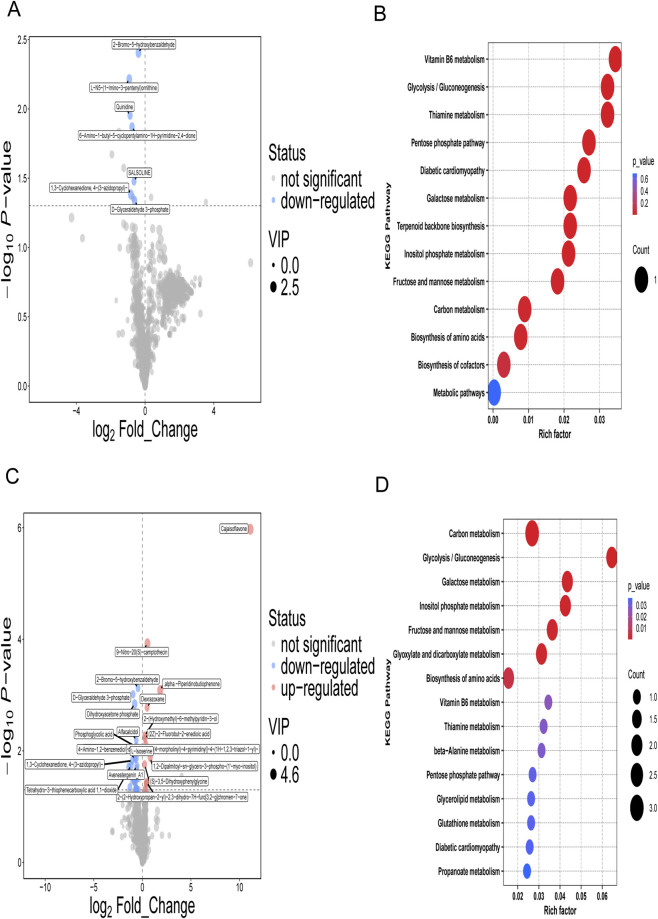
Metabolic profile in red blood cell (RBC) from 30 β-thalassemia major (β-TM) patients at baseline and incubate with SNH-119014. **(A)** Volcano plot of differentially abundant metabolites between β-TM and β-TM incubated with SNH-119014. **(B)** KEGG enrichment analysis of differentially abundant metabolites between β-TM and incubated with SNH-119014. **(C)** Volcano plot of differentially abundant metabolites between β-TM and incubated with AG-348. **(D)** KEGG enrichment analysis of differentially abundant metabolites between β-TM and incubated with AG-348. Note: The dot size was proportional to the VIP value, with larger dots representing higher VIP scores. Significantly upregulated, downregulated, and non-significant metabolites are depicted in red, blue, and gray, respectively. The rich factor represents the proportion of differential metabolites mapped to a given pathway relative to the total number of metabolites identified in that pathway. A higher value indicates greater enrichment of that pathway in the comparison.

To evaluate the specific metabolic impact of SNH-119014, we analyzed the metabolomic profiles of β-TM RBCs following compound incubation. Unsupervised principal component analysis (PCA) showed limited separation between baseline and treatment groups for both compounds (Figures 5A,B). However, supervised orthogonal partial least squares-discriminant analysis (OPLS-DA) revealed distinct metabolic remodeling induced by each activator. SNH-119014 prompted a moderate shift from baseline (Figures 5C,E,G), whereas AG-348 elicited a more pronounced separation (Figures 5D,F,H). A total of 1,219 differential metabolites were identified in RBCs after incubation with SNH-119014 compared to baseline, which included the glycolytic intermediate D-glyceraldehyde 3-phosphate (Figure 6A). In contrast, AG-348 modulated 34 differential metabolites, with the most downregulated species being dihydroxyacetone phosphate and phosphoglycolic acid, both critical to glycolytic flux (Figure 6B).

KEGG pathway enrichment analysis highlighted the most significantly altered metabolic pathways. Treatment with SNH-119014 significantly enriched pathways including Vitamin B6 metabolism, Glycolysis/Gluconeogenesis, and the Pentose Phosphate Pathway (PPP), among others (Figure 6B). AG-348 affected a broader set of pathways, with top enrichments in Glycolysis/Gluconeogenesis, Fructose and mannose metabolism, Vitamin B6 metabolism, PPP, and Glutathione metabolism (Figure 6D). Critically, both compounds significantly altered glycolysis/gluconeogenesis and the PPP.

To determine whether SNH-119014 treatment partially reverses the lipid abnormalities characteristic of β-TM, we specifically examined the glycerophospholipid profiles. Compared to healthy controls, β-TM RBCs exhibited 86 differentially abundant glycerophospholipids. Following *ex vivo* incubation with SNH-119014, this number was reduced to 57.

### Modulation of glycolytic flux by SNH-119014

**FIGURE 7:**
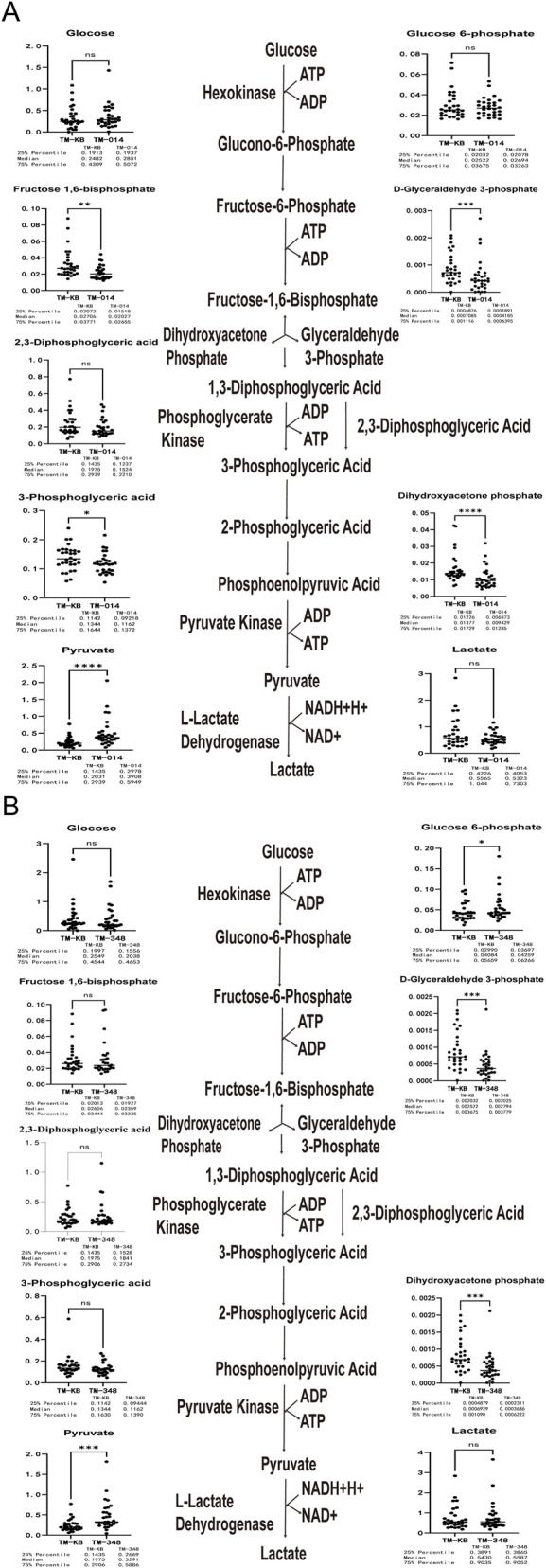
SNH-119014 alters the glycolysis pathway of red blood cell (RBC) in β-thalassemia major (β-TM). **(A)** Effect of SNH-119014 on metabolites in the Glycolysis Pathway within RBC. **(B)** Effect of AG-348 on metabolites in the glycolysis pathway within RBCs. Note: NS stands for Not Significant; * indicates p < 0.05; ** indicates p < 0.01.

To delineate the effects of PK activation on glycolytic metabolism, we first compared the glycolytic pathway metabolites between β-TM and HC RBCs, as shown in Supplementary Figure S1. We then mapped the glycolytic flux in β-TM RBCs following compound treatment. Compared with the RBC of β-TM without incubating SNH-119014, the contents of upstream metabolites glucose and glucose-6-phosphate of the glycolytic pathway in RBCs of β-TM incubated with SNH-119014 were similar, and the glycolytic intermediate product fructose-1,6-diphosphate, glyceraldehyde-3-phosphate, 3-phosphoglyceric acid, and phosphodihydroxyacetone decreased, while pyruvate downstream of the pyruvate kinase action site increased (Figure 7A). We evaluated the effect of AG-348 on the metabolism of RBC in β-TM on the glycolytic pathway (Figure 7B). Compared with RBC of β-TM without incubating AG-348, the contents of upstream metabolites glucose-6-phosphate of the glycolytic pathway in mature red blood cells of β-TM incubated with AG-348 were higher, and the glycolytic intermediate product the glycolytic intermediate products glyceraldehyde-3-phosphate and phosphodihydroxyacetone decreased, while pyruvate downstream of the pyruvate kinase action site increased. Collectively, the pyruvate accumulation indicates an accelerated terminal glycolysis upon PK activation by both compounds.

### Amelioration of oxidative stress in erythroid precursors by SNH-119014

During erythropoiesis, the dominant pyruvate kinase isoform shifts from PKM2 in precursors to PKLR in mature red blood cells (Nijhof et al., 1984) (Supplementary Figures 2A–D). Therefore, to comprehensively evaluate the therapeutic potential of SNH-119014, it was necessary to examine its effects specifically on erythroid precursor cells. Given the critical role of oxidative stress in the ineffective erythropoiesis of thalassemia (Voskou et al., 2015), we assessed the impact of SNH-119014 on erythroid precursors derived from β-TM patients.

**FIGURE 8:**
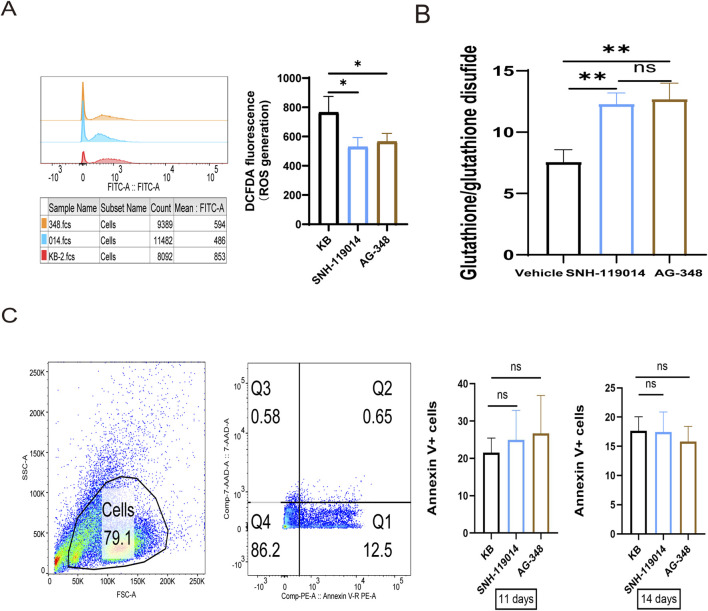
SNH-119014 alleviates oxidative stress of red blood cell (RBC) without compromising its differentiation. **(A)** ROS levels in erythroid precursors from β-TM treated with vehicle (KB) or compounds. **(B)** GSH/GSSG ratio in erythroid precursors from β-TM treated with vehicle or SNH-119014, or AG-348. **(C)** No difference in apoptosis rate of erythroid precursor cells of βthalassemia major after incubation with SNH-119014 or AG-348. Note: Experiments were performed in triplicate. Data are mean ± SD (n = 3).

In CD34^+^ derived erythroid precursors, both SNH-119014 and AG-348 (5 µM) significantly alleviate oxidative stress. On day 11 of culture, treatment with either compound reduced intracellular ROS levels (Figure 8A) and increased the reduced-to-oxidized glutathione (GSH/GSSG) ratio (Figure 8B) compared to the vehicle control. These coordinated changes demonstrate a potent alleviation of oxidative stress. To determine whether PK activation influences erythroid maturation, we monitored the surface expression of differentiation markers CD235a and CD71 throughout the culture period. No significant differences were observed between cells treated with SNH-119014, AG-348, or vehicle (Supplementary Figure S3). Similarly, the proportion of Annexin V^+^ apoptotic cells remained comparable across all groups (Figure 8C). These results indicate that SNH-119014 does not alter the maturation process or survival of β-TM erythroid precursors under these conditions.

## Discussion

This study demonstrates that SNH-119014, a novel allosteric activator of PK, exerts multifaceted beneficial effects on the pathophysiology of β-TM. We show that it directly activates PKLR, reprograms RBC metabolism to enhance glycolytic flux and pentose phosphate pathway activity, and alleviates oxidative stress in erythroid precursors. These findings collectively suggest that SNH-119014 holds therapeutic potential by targeting the core metabolic dysfunctions in β-TM.

Adequate ATP levels are crucial for erythrocyte integrity, supporting calcium homeostasis via the plasma membrane Ca^2+^ ATPase to prevent dehydration through the Gardos channel, and maintaining membrane deformability to avoid phosphatidylserine exposure and subsequent erythrophagocytosis (Park et al., 2010; van Dijk et al., 2023; Gallagher, 2017). In this study, our finding that SNH-119014 elicits a mean 1.29-fold increase in ATP levels in β-TM RBCs (range: 1.12–1.47) is physiologically significant and aligns with a previously reported effect of AG-348 (Rab et al., 2019). It is noteworthy that although SNH-119014 exhibited a higher AC_50_ (28.90 nM) than AG-348 (13.71 nM) in the biochemical assay, its substantially greater maximal efficacy (Emax: 949.9% vs. 749.4%) likely compensates at the saturating concentrations used in cellular assays, resulting in the comparable ATP elevation observed. Notably, this ATP-enhancing effect of SNH-119014 was consistent across RBCs derived from patients with a broad spectrum of β-thalassemia genotypes. However, as direct functional assays were not performed in the present study, the extent to which the observed ATP elevation translates into improved membrane integrity or RBC survival *ex vivo* remains to be established.

Our metabolomic profiling delineated a distinct metabolic signature in β-TM RBCs, characterized most prominently by alterations in glycerophospholipids. This likely reflects membrane lipid peroxidation damage (Farmakis et al., 2021), a known consequence of oxidative stress in thalassemia. Concurrently, elevated carnitine species suggest a compensatory activation of the Lands cycle for membrane repair, as carnitines are utilized in RBCs to mitigate lipid peroxidation (Van Dijk et al., 2024). The observed pyruvate accumulation is a direct result of SNH-119014 activating PK, thereby enhancing the conversion of phosphoenolpyruvate (PEP) to pyruvate.

KEGG pathway analysis confirmed that glycolysis/gluconeogenesis was among the most significantly altered pathways following treatment with either compound.

Furthermore, both compounds modulated the pentose phosphate pathway (PPP), which enhances NADPH regeneration to support glutathione-mediated antioxidant defenses (Orrico et al., 2023), a mechanism that likely contributes to the hemoglobin increase observed in clinical trials of AG-348 (Kuo et al., 2022). Collectively, these findings lead us to hypothesize that the therapeutic benefit of PK activators in anemia is mediated by concurrent improvement in ATP-dependent membrane stability and glutathione antioxidant capacity. Furthermore, glutathione metabolism was another top-ranked KEGG pathway associated with AG-348 treatment. However, this effect was not observed in RBC incubated with SNH-119014. The mechanisms underlying these differences in metabolomic profiles and their pathophysiological and therapeutic implications remain to be elucidated.

We also assessed the impact of SNH-119014 on erythroid precursors, given the contribution of ineffective erythropoiesis to β-TM anemia. In CD34^+^ derived erythroid precursors from β-TM patients, SNH-119014 significantly alleviated oxidative stress by reducing ROS levels and elevating the GSH/GSSG ratio, demonstrating direct protective efficacy at the progenitor level.

Notably, our findings regarding differentiation differ from some prior reports. While earlier studies indicated that AG-348 promotes erythroid differentiation and reduces apoptosis in thalassemic models (Matte et al., 2021), we observed no significant effect of either SNH-119014 or AG-348 on the surface expression of erythroid markers (CD71^+^/CD235a^+^) or on apoptosis rates in our culture system. This lack of pro-differentiation effect aligns with observations in some models of pyruvate kinase deficiency, suggesting that the primary benefit of PK activation in this context may be the correction of oxidative stress rather than direct stimulation of maturation. Further research is needed to clarify the specific conditions under which PK activators influence erythroid differentiation.

## Limitations and future perspectives

While this study provides *ex vivo* and cellular evidence supporting the pharmacological potential of SNH-119014, it is important to acknowledge its limitations. Our data demonstrate that SNH-119014 enhances ATP production and reduces oxidative stress in both RBCs and erythroid precursors, however evidence linking these metabolic improvements to functional outcomes is currently lacking. Future studies employing thalassemic animal models are essential to validate these promising *ex vivo* findings, assess hematological improvement, and further elucidate the *in vivo* mechanisms of action.

Additionally, the age and sex distribution differed between the β-TM patient group and healthy controls (Table 1). Formal statistical adjustment for these covariates was not performed, and this should be considered when interpreting the disease-specific metabolic signature reported in this study.

In conclusion, our data demonstrate that SNH-119014 activates PK, enhances cellular ATP production, and alleviates oxidative stress in erythroid cells from patients with β-TM. These findings reveal, for the first time, that SNH-119014 exhibits a favorable pharmacodynamic profile similar to AG-348 in an *ex vivo* model, underscoring its potential as a therapeutic agent for thalassemia.

## Data Availability

The metabolomics datasets generated and analyzed during this study are publicly available in the Metabolomics Workbench repository under Project ID PR002900 (Study ID ST004602). The original contributions presented in the study are also included in the article/Supplementary Material. Further inquiries can be directed to the corresponding authors.

## Supplementary material

SUPPLEMENTARY FIGURE S1 Comparison of glycolytic pathway metabolites in red blood cell of 30 β-thalassemia major patients and 30 healthy controls.

SUPPLEMENTARY FIGURE S2 Red blood cells (RBCs) mainly express PKLR, while erythroid precursors mainly express PKM2. Representative Western blot bands and relative quantification of target protein expression. **(A, B)** PKLR was mainly expressed in RBCs from β-thalassemia major patients (β-TM) and healthy volunteers (HC). Experiments of each sample were performed in triplicate. Data are mean ± SD (n = 3). **(C, D)** PKM2 was mainly expressed in erythroid precursors from β-TM and HC. Experiments of each sample were performed in triplicate. Data are mean ± SD (n = 3). (E) SNH-119104 increases ATP levels of erythroid precursors. Experiments of each sample were performed in triplicate. Data are mean ± SD (n = 3). Note: **** indicates p < 0.0001.

SUPPLEMENTARY FIGURE S3 SNH-119014 did not affect the differentiation process of erythroid precursor cells. **(A)** Flow cytometric analysis of CD235a and CD71 expression on erythroid precursors from patients with β-thalassemia major. Experiments of each sample were performed in triplicate. Data are mean ± SD (n = 14). **(B)** Flow cytometric analysis of CD235a and CD71 expression on erythroid precursors from healthy controls. Experiments of each sample were performed in triplicate. Data are mean ± SD (n = 4).

SUPPLEMENTARY FIGURE S4 The relative ATP levels in erythroid precursors varies different Concentration of compounds. **(A)** ATP levels in erythroid precursors of β-Thalassemia Major. **(B)** ATP levels in erythroid precursors of healthy controls.
