# Supplementary material for: SNH-119014, a novel pyruvate kinase activator, enhances ATP production and reduces oxidative stress in erythroid cells from patients with β-thalassemia major
Source: Front Pharmacol. 2026 Apr 23;17:1719328. doi: 10.3389/fphar.2026.1719328 (PMC13149418; doi:10.3389/fphar.2026.1719328)
Supplement: Supplementary file 1 [file DataSheet1.pdf]

SUPPLEMENTAL FIGURES

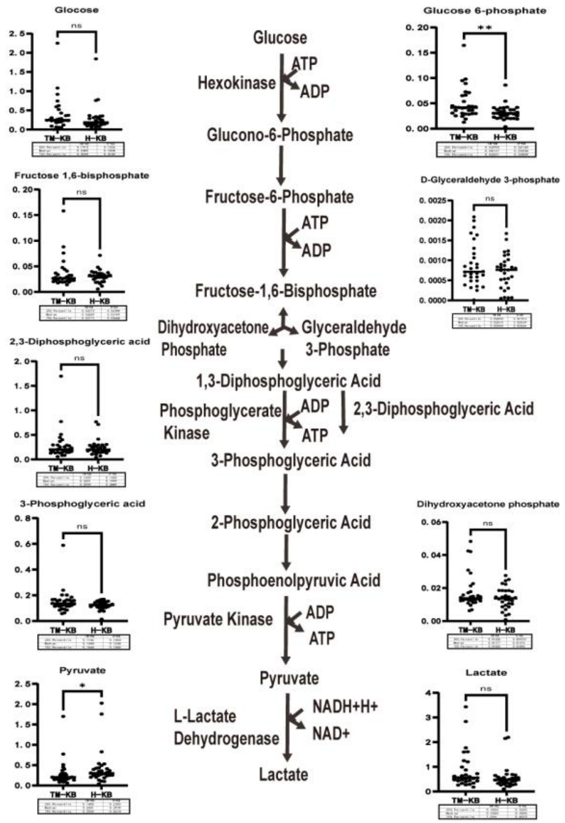

Supplemental Figure 1. Comparison of glycolytic pathway metabolites in red blood cell of 30 β-thalassemia major patients and 30 healthy controls.

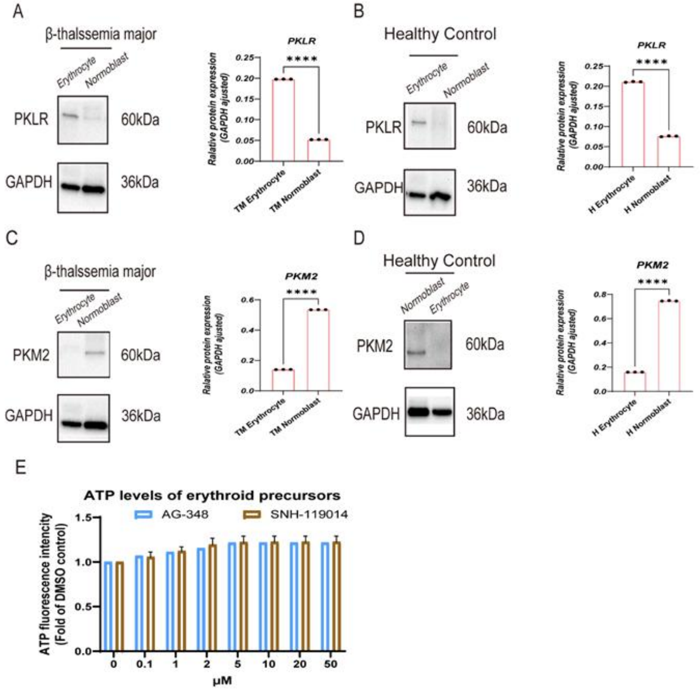

Supplemental Figure 2. Red blood cells (RBCs) mainly express PKLR, while erythroid precursors mainly express PKM2. Representative Western blot bands and relative quantification of target protein expression. (A, B) PKLR was mainly expressed in RBCs from β-thalassemia major patients (β-TM) and healthy volunteers (HC). Experiments of each sample were performed in triplicate. Data are mean ± SD (n=3). (C, D) PKM2 was mainly expressed in erythroid precursors from β-TM and HC. Experiments of each sample were performed in triplicate. Data are mean ± SD (n=3). (E) SNH-119104 increases ATP levels of erythroid precursors. Experiments of each sample were performed in triplicate. Data are mean ± SD (n=3). Note: **** indicates $p < 0.0001$.

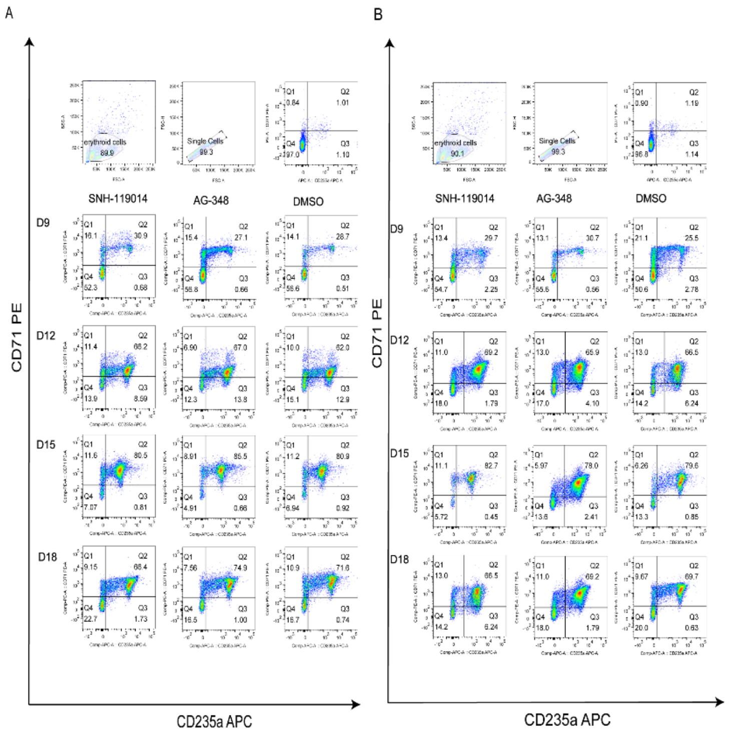

Supplemental Figure 3. SNH-119014 did not affect the differentiation process of erythroid precursors. (A) Flow cytometric analysis of CD235a and CD71 expression on erythroid precursors from patients with β-thalassemia major. Experiments of each sample were performed in triplicate. Data are mean ± SD (n=14). (B) Flow cytometric analysis of CD235a and CD71 expression on erythroid precursors from healthy controls. Experiments of each sample were performed in triplicate. Data are mean ± SD (n=4).

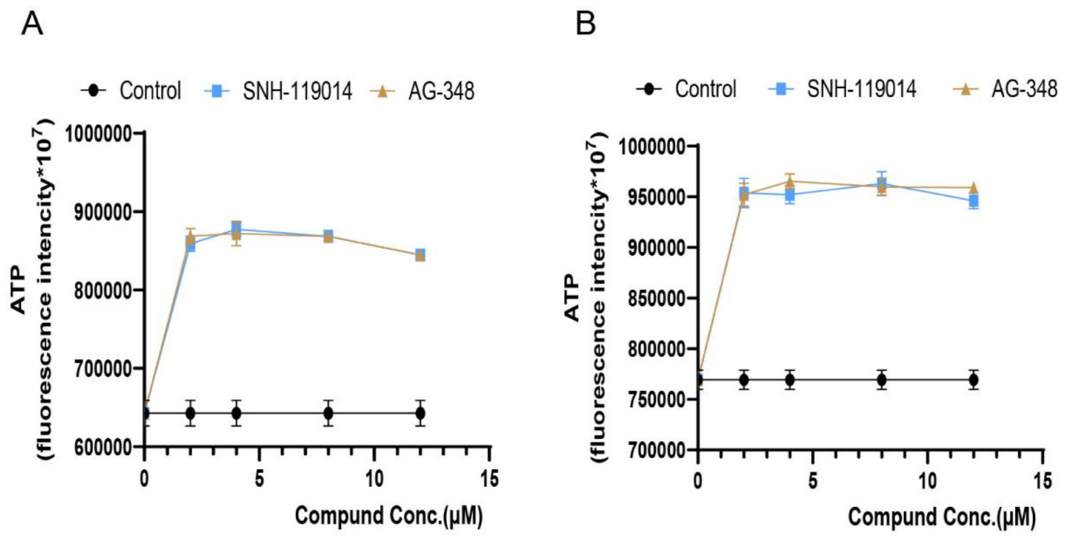

Supplemental Figure 4. The relative ATP levels in erythroid precursors varies different Concentration of compounds. (A) ATP levels in erythroid precursors of β-Thalassemia Major. (B) ATP levels in erythroid precursors of healthy controls.

SUPPLEMENTAL TABLES

Supplemental Table S1. ATP levels in Red Blood Cells of β-Thalassemia Major Incubated with SNH-119014 at Different Concentrations and Durations

Concentration of SNH-119014 (μM)	0 hour	2 hour	4 hour	8 hour	12 hour
0μmol/L	642764±16510	642754±16510	642750±16514	642633±16522	642600±16522
0.5μmol/L	642764±16510	751735±11609 *	751322±11647 *	751633±11545 *	742600±14340 *
1μmol/L	642764±16510	792288±24897 *#	790154±24628 *#	792145±24957 *#	750656±10143 *
3μmol/L	642764±16510	860707±7678 *#	858193±2984 *#	858833±7757*#	845618±3019 *#
10μmol/L	642764±16510	860712±7680 * #	858203±2979 * #	858870±7759 *#	845588±3033 *#
50μmol/L	642764±16510	860736±7679 *	858224±2992 *	858870±7759 *	845609±3035 *
100μmol/L	642764±16510	860718±7678 *	858210±2988 *	858843±7758 *	845583±3032 *

Notes: Data are presented as mean ± SD. * represents ATP levels of different SNH-119014 concentration vs. 0 μmol/L under the same incubation times; # represents ATP levels of different incubation times vs. 0 μmol/L under the same concentration.

Supplemental Table S2. ATP levels in Red Blood Cells of β-Thalassemia Major Incubated with AG-348 at Different Concentrations and Durations

Concentration of AG-348 (μM)	0 hour	2 hour	4 hour	8 hour	12 hour
0μmol/L	642764±1610	642764±16510	642764±1651	642358±16520	642348±16500
0.5μmol/L	642764±16510	735586±18542ns	737568±22548 *	735821±14234 *	734835±41034 *
1μmol/L	642764±16510	770160±12558 *	767188±19698 *	773318±30263 *	764388±22019 *
3μmol/L	642764±16510	860244±31791 *	865154±43734 *	863528±8921 *	851303±12861 *
10μmol/L	642764±16510	859116±9700 *	88800715736 *	868347±3912 *	844941±4647 *
50μmol/L	642764±16510	870292±24401 *	864374±28060 *	854422±12290 *	846482±10403*
100μmol/L	642764±16510	867757±9199 *	877893±6911 *	854422±31360 *	839528±6605 *

Notes: Data are presented as mean ± SD. * represents ATP levels of different AG-348 concentration vs. 0μmol/L under the same incubation times; # represents ATP levels of different incubation times vs. 0μmol/L under the same concentration.

Supplemental Table S3. ATP levels in Red Blood Cells of healthy controls Incubated with SNH-119014 at Different Concentrations and Durations

Concentration of SNH-119014 (μM)	0 hour	2 hour	4 hour	8 hour	12 hour
0μmol/L	769274±9505	769274±9505	769219±9500	769118±9522	769094±9405
0.5μmol/L	769274±9505	823493±1931 *	826619±6653 *	838318±26597 *	834851±16909 *
1μmol/L	769274±9505	921772±14594 *	920791±13663 *	925126±12770 *	909106±22499 *
3μmol/L	769274±9505	954496±9120 *	958725±10856 *	953907±14495 *	936531±6636 *
10μmol/L	769274±9505	953963±14553 *	952010±9018 *	963160±11691 *	953160±11691 *
50μmol/L	769274±9505	951869±15003 *	947565±23771 *	951630±28041 *	938297±24446 *
100μmol/L	769274±9505	946880±28595 *	947166±16766 *	952921±9389 *	939587±13255 *

Notes: Data are presented as mean ± SD. * represents ATP levels of different SNH-119014 concentration vs. 0 μmol/L under the same incubation times; # represents ATP levels of different incubation times vs. 0 μmol/L under the same concentration.

Supplemental Table S4. ATP levels in Red Blood Cells of healthy controls Incubated with AG-348 at Different Concentrations and Durations

Concentration of AG-348 (μM)	0 hour	2 hour	4 hour	8 hour	12 hour
0μmol/L	769274±9505	767664±9005	768871±6505	749444±8705	739374±7505
0.5μmol/L	769274±9505	823493±1931 *	826619±6653 *	834984±17106 *	834851±16909 *
1μmol/L	769274±9505	925105±10290 *	915356±12654 *	928460±7146 *	912439±19155 *
3μmol/L	769274±9505	949361±16243 *	954650±9598 *	955241±13395 *	938154±9363 *
10μmol/L	769274±9505	952114±11383 *	965449±7216 *	959827±7993 *	959051±4392 *
50μmol/L	769274±9505	953464±16499 *	947565±23771 *	944964±21807 *	943034±16263 *
100μmol/L	769274±9505	943488±20553 *	947300±16772 *	949587±3615 *	942764±9527 *

Notes: Data are presented as mean ± SD. * represents ATP levels of different AG-348 concentration vs. 0μmol/L under the same incubation times; # represents ATP levels of different incubation times vs. 0μmol/L under the same concentration.
